# Two Distinct Myeloid Subsets at the Term Human Fetal–Maternal Interface

**DOI:** 10.3389/fimmu.2017.01357

**Published:** 2017-10-25

**Authors:** Maria Laura Costa, Michelle L. Robinette, Mattia Bugatti, Mark S. Longtine, Bryanne N. Colvin, Erica Lantelme, William Vermi, Marco Colonna, D. Michael Nelson, Marina Cella

**Affiliations:** ^1^Department of Obstetrics and Gynecology, Washington University School of Medicine, St Louis, MO, United States; ^2^Department of Obstetrics and Gynecology, University of Campinas (UNICAMP), Campinas, Brazil; ^3^Department of Pathology and Immunology, Washington University School of Medicine, St Louis, MO, United States; ^4^Department of Molecular and Translational Medicine, Section of Pathology, University of Brescia, Brescia, Italy; ^5^Department of Pediatrics, Washington University School of Medicine, St Louis, MO, United States

**Keywords:** placenta, antigen-presenting cells, inflammation, tolerance, pregnancy

## Abstract

During pregnancy, immune cells infiltrate the placenta at different stages of fetal development. NK cells and macrophages are the most predominant cell types. These immune cells play pleiotropic roles, as they control spiral artery remodeling to ensure appropriate blood supply and maintain long-term tolerance to a true allograft; yet, they must be able to mount appropriate immune defenses to pathogens that may threaten the fetus. Whether the same cell type accomplishes all these tasks or if there are dedicated subsets remains controversial. Here, we identify and characterize two distinct subsets of myeloid cells that differ in their pro-inflammatory/regulatory capacity. While one subset predominantly produces the immune-modulating cytokine IL-10, the second subset has superior capacity to secrete pro-inflammatory mediators, such as IL-1β and IL-6. The putative regulatory myeloid cells also express high levels of inhibitory receptors and their ligands, including programmed cell death 1 (PD1) ligands. Importantly, a large fraction of CD8 and CD4 cells in normal term human placenta are PD1 positive, suggesting that the PD1/PD1 ligands axis might be critical to maintain tolerance during pregnancy.

## Introduction

Pregnancy is a unique situation during which a truly hemi-allogeneic tissue is well protected from the attack of immune cells that would otherwise have the task of defending “self” from “foreign.” Still, maternal cells must be capable of mounting some protective response against bacteria or pathogens that may harm the fetus (1). Therefore, there must be mechanisms in place that tightly regulate the immune landscape and compromise tolerance with defense during pregnancy (2).

The placenta is heavily infiltrated from immune cells throughout pregnancy (3, 4). The two most prominent cell types in the placenta are NK cells and macrophages, both of which exert distinct and cooperative functions. Collectively, they are involved in trophoblast invasion, spiral artery remodeling and angiogenesis, removal of apoptotic cells, and protection of the implant from infections (3, 5).

To understand how tolerance is enforced in human placenta, we evaluated dendritic cell (DC) representation in this tissue, as DCs are key to maintain peripheral tolerance (6). Surprisingly, we found very few DCs in the basal plate (BP) of normal term human placenta. We instead directed our attention to a subset of CD14^+^ MHC classII^high^ myeloid cells, most likely macrophages, that based on functional studies and unbiased gene array profiling appear to have tolerogenic and regulatory properties.

## Materials and Methods

### Collection of Placental Tissue

This study was carried out in accordance with the recommendations of Washington University School of Medicine IRB committee with written informed consent from all subjects. All subjects gave written informed consent in accordance with the Declaration of Helsinki. The protocol was approved by the Institutional Review Board of the Washington University School of Medicine, St. Louis, MO, USA. Placentas were obtained from singleton, term (37–40 weeks), uncomplicated gestations delivered by scheduled cesarean section under conduction of anesthesia without labor or delivered vaginally after spontaneous labor, with delivery in 6 h. Placentas were kept at 4°C until sampling, with an average time between delivery and sampling of 60 min (range 30–120 min). The placenta was then submitted to random sampling of the BP (maternal–fetal interface), according to recommended procedure for placental sample collection (7). Five to ten *en face* sections of BP tissue were taken with ~1.5 cm^2^ surface area and <0.5 cm thickness, avoiding areas of visible infarcts or calcifications.

### Isolation of Cells from the BP, Flow Cytometry, and Cell Sorting

Basal plate tissue was finely minced with scissors, digested with collagenase D (1 mg/ml, Roche) for 60 min at 37°C in RPMI medium with 10% FBS (Hyclone) and pressed through a series of strainers to yield single cells. For identification of DC populations, single cells were stained with a combination of lineage markers (CD3, CD19-PerCP-Cy5.5 eBioscience; CD16, CD56, and CD14-PerCP-Cy5.5 Biolegend), CD45-AlexaFluor-700 (Biolegend), HLA-DR-APC (BD), ILT3-PE-Cy7 (Biolegend), ILT1-PE (eBioscience), CD303-FITC (Miltenyi), and CD1c-Brilliant Violet 510 (Biolegend). Cells were acquired on a LSR-Fortessa (BD) and data analyzed with the FlowJo software. For FACS sorting experiments CD14 cells were pre-enriched by magnetic purification (CD14 microbeads, Miltenyi), stained with CD14-FITC (Beckman Coulter) and HLA-DR-APC (BD), and separated on FACS-AriaII (BD). For morphological analysis, CD14^+^MHCII^high^ and CD14^+^ MHCII^low^ cells were immobilized on slides by cytospin and stained with a Hema 3 stain set. Additional antibodies used for flow cytometry analysis: immunoglobulin-like transcript (ILT) 2, CD40, CD54, CD80, CD50, CD89-PE (Beckman Coulter); ILT4-APC (eBioscience); ILT5-PE (eBioscience); CXCR4-PE (BD); CD91-eFluor660 (eBioscience); CD206-Brilliant Violet 421; CRACC-PE, CD180-PE, CLEC4A-PE (Biolegend); DQ-FITC (eBioscience); CD9-FITC, CD35-FITC (Beckman Coulter); IDO-PE (eBioscience); mouse IgG1 Control-PE (eBioscience); CLEC4A-PE (Biolegend); CD3-Brilliant Violet 605 (Biolegend); PD1-Brilliant Violet 421 (Biolegend); CD4-APC (eBioscience); CD8-PerCP-Cy5.5 (Biolegend); CD8-APC (eBioscience); and CD4-APC-eFluor-780 (eBioscience).

### Statistical Analysis

Statistics were calculated as indicated in figure legends using the Prism7 software.

### Histology

Formalin-fixed paraffin-embedded tissue blocks used for this study were retrieved from the tissue bank of the Department of Pathology (ASST-Spedali Civili di Brescia, Brescia, Italy). Tissues used for the analysis included normal placental tissue at the first (five cases) and third (ten cases) trimester. Four-micron-thick tissue sections were used for immunohistochemical staining. Primary antibodies included anti-CD14 (1:50, mouse, clone 7, Leica); anti-CD163 (1:50, mouse, clone 10D6, Neomarkers); and anti-HLA-DP,DQ,DR(1:500, mouse, clone CR3/43, DAKO). The reaction was revealed using Novolink Polymer (Leica Microsystems) followed by DAB. Microphtalmia-associated transcription factor (MITF) (1:50, mouse, clone D5, DAKO) was visualized using Mach 4 MR-AP (Biocare Medical), followed by Ferangi Blue (Biocare Medical) as chromogen.

### GPNMB Staining by Immunofluorescence

Cells were FACS sorted and immobilized on slides by cytospin. They were then dried overnight and fixed for 15 min in methanol at −20°C. After blocking for 1 h at room temperature with PBS containing 5% FBS, cells were incubated with biotinylated anti-GPNMB (R&D) (1:100) overnight at 4°C, followed by 1:200 streptavidin-PE (eBioscience) and DRAQ5 1:500 for DNA staining. Images were obtained at 600X magnification using a Nikon E800 confocal microscope. Three different placentas were sorted and cells placed by cytospin on two slides per placenta, and 10 random pictures of each slide were photographed. Intensity of positive cells for GPNMB per field was quantified using ImageJ software (NIH), by considering the mean gray value within each cell, compared to the average background.

### Transcriptome Analysis and Real-time PCR

For gene expression microarrays, RNA from sorted populations (*n* = 4 HLA-DR^high^ replicates and *n* = 3 HLA-DR^low^ replicates) was prepared using the RNeasy micro kit (Qiagen), following manufacturer instructions. Gene array data analysis and ingenuity pathway analysis (IPA) were performed as previously described (8). The Affymetrix Human Gene (v.1.0) ST array platform was used (8). Microarrays have been deposited in GEO under accession number GSE104224.

RNA isolation, cDNA generation, and quantitative real-time PCR (qRT-PCR) were done as previously described (9), except using a total reaction volume per well of 10 µl. To verify amplification of a single product with the appropriate melting temperature, dissociation curves were evaluated for all reactions. RNA expression levels were normalized to parallel reactions with primers specific for *GAPDH*. The fold increase gene expression in experimental relative to control conditions was determined by utilizing the 2^−ΔΔCt^ method (10).

Primer list: *GPNMB* 5′-TAAACCTTGAGTGCCTGCGT, 3′-TGAAATCGTTTGG CGGCATC; *UBD* 5′-TCTCTGGTTTCTGGCCCCTT, 3′-CGGAACGGACA TGCACACAG; *EBI3* 5′-AGAGCACATCATCAAGCCCG, 3′-CAGCTCCCTGACGCTTGTAA; *CD80* 5′-GGGGAAATGTCGCCTCTCTG, 3′-GTGGATTTAGTTTCACAGCTTGC; *IDO1* 5′-GATGTGGGCTTTGCTCTACC, 3′-GCTTCCCATTCTCAATCAGC; *MITF*: 5′-TGTGACTGAACCAACTGGCACTTAC, 3′-TGCTCCGCCTGCTACTCGTT; *S100A12*: 5′-CACTGCTGGCTTTTTGCTGT, 3′-AATGCCCCTTCCGAACTGAG; *F5*: 5′-CACGTGGTTCACTTTCACGG, 3′-AATGAAC CAGGCAGAAGGGG; and *GAPDH*: 5′-CCTGGTATGACAACGAATTT, 3′-AGTGAGGGTCTCTCTCTTCC.

### Tissue Culture for Cytokine Quantification

CD14^+^ HLA-DR^high^ and HLA-DR^low^ cells were plated in identical numbers (10^5^ cells/well in duplicate) in 200 µl of complete RPMI medium in wells of a 96 flat bottom plate. Cells were immediately stimulated with TLR agonists Resiquimod (5 µg/ml; inVivogen); LPS (100 ng/ml, Ultrapure LPS K12; inVivogen); PolyIC (50 µg/ml; Amersham), Pam3CSK4 (50 ng/ml; inVivogen), and CpG 1826 (6 µg/ml; Qiagen). Supernatants were collected after 18 h and cytokines quantified by Cytometric Beads Arrays (Human Inflammation, BD).

### Collection of Term and Pre-Term Placentas for GPNMB Quantification

A case–control study was also included, under local Ethical approval. Subjects were enrolled and consented in the Women and Infant’s Health Specimen Consortium (WHISC). The study was conducted within the Washington University Medical School and Barnes Jewish Hospital in Saint Louis, MO, USA from September 2014 to April 2016. Samples and demographic data collection were de-identified. Placenta was obtained, as previously described in tissue preparation, from singleton gestation of normotensive term (≥37–41 weeks) gestations (*n* = 20), term pre-eclampsia (*n* = 20), and pre-term pre-eclampsia with severe features (≤34 weeks) (*n* = 16). Pre-eclampsia was diagnosed as new onset of hypertension and either proteinuria or end-organ dysfunction after 20 weeks of gestation in a previously normotensive woman (11). Pre-eclampsia with severe features was defined by the presence of one or more of the following: unrelieved headache, hepatic abnormality, severe blood pressure elevation, thrombocytopenia, progressive renal insufficiency, and pulmonary edema (11). Women with multi-fetal gestations, known medical conditions, including chronic hypertension, diabetes mellitus, renal or collagen vascular disease, and fetal chromosomal abnormalities, were excluded.

Random samples of tissue from the BP were retrieved from delivered placentas, frozen in liquid nitrogen and stored at −80°C. RNA was extracted using TRIzol, cDNA prepared from 0.5 µg of total RNA and qRT-PCR carried out as described earlier.

## Results

### DC Subsets in Term Placenta Are Poorly Represented Compared to Peripheral Blood

**Figure 1:**
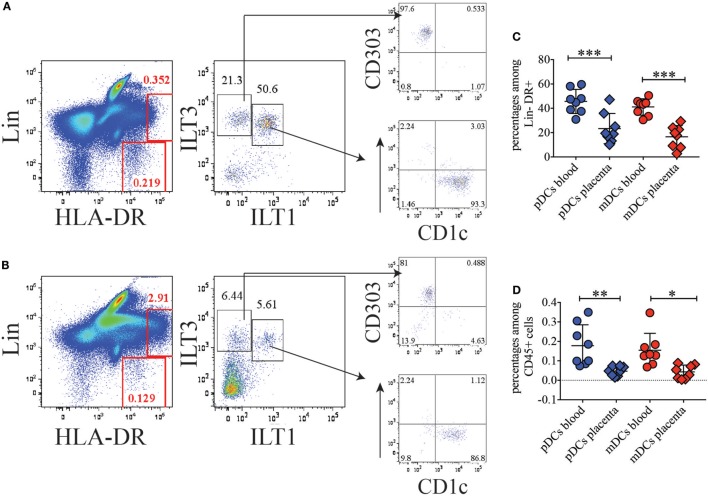
Conventional dendritic cell (DC) subsets are poorly represented in basal plate tissue of normal term placentas. **(A)** Identification of conventional DC subsets in blood and **(B)** basal plate tissue of normal term placenta. DC subsets were identified within the Lineage negative HLA-DR^+^ population as ILT3^+^ILT1^−^ plasmacytoid DCs (pDCs), which express BDCA2 (CD303), and as ILT3^+^ILT1^+^ myeloid DCs (mDCs), which express CD1c. One representative of eight individual donors for blood and placenta is shown. **(C,D)** Percentages of pDCs and mDCs in blood and placenta among lineage negative HLA-DR^+^ **(C)** or among total CD45^+^ **(D)** cells. Significance was calculated by ordinary one-way ANOVA Tukey’s multiple comparison test. *p < 0.05, **p < 0.005, and ***p < 0.0005.

To investigate the nature and abundance of DCs in the BP of normal term human placenta compared to peripheral blood, we took advantage of our previous staining criteria to identify DC subsets in circulating blood (12). After collagenase extraction, we stained placenta cells with a mixture of CD45, to identify hematopoietic cells, lineage (Lin) markers (CD3, CD19, CD56, CD14, and CD16) to exclude T, B, NK, monocytes, and granulocytes, respectively, and HLA-DR. Among CD45^+^, Lin-negative HLA-DR^+^ cells, we distinguished plasmacytoid DCs (pDCs) and myeloid DCs (mDCs) based on the expression of ILTs, ILT3 (CD85K, LILRB4) and ILT1 (CD85H, LILRA2). In blood, ILT3 single positive pDCs homogenously expressed CD303 (BDCA2), while ILT3/ILT1 double positive cells uniformly expressed CD1c (Figure 1A). However, in placenta, DC subsets were less prominent than in blood (Figure 1B). Additionally, the frequency of pDCs and mDCs among Lin-negative HLA-DR^+^ cells was significantly lower in the BP tissue (pDCs: 35.9–10.1%; mDCs: 29.2–2.7%) than in blood (pDCs: 59.6–30.9%; mDCs: 50.5–30.5%) (Figure 1C). Frequencies of pDCs and mDCs in placenta were also significantly lower when percentages of the respective DC subsets were calculated among total CD45^+^ hematopoietic cells (Figure 1D) (placenta: pDCs: 0.075–0.015%; mDCs: 0.072–0.039%; blood: pDCs: 0.35–0.07%; mDCs: 0.18–0.08%).

During these experiments, we noticed a population of Lin^+^ cells in placenta, which exhibited high expression of HLA-DR and was poorly represented in blood (Figures 1A,B). We reasoned that high expression levels of MHC class II might be linked to antigen processing and presentation. Therefore, we further sought to better characterize this cell subset.

### HLA-DR^high^ Cells Are a CD14^+^ Myeloid Cell Subset That Produces Higher Levels of IL-10 upon TLR Stimulation than HLA-DR^low^ Cells

**Figure 2:**
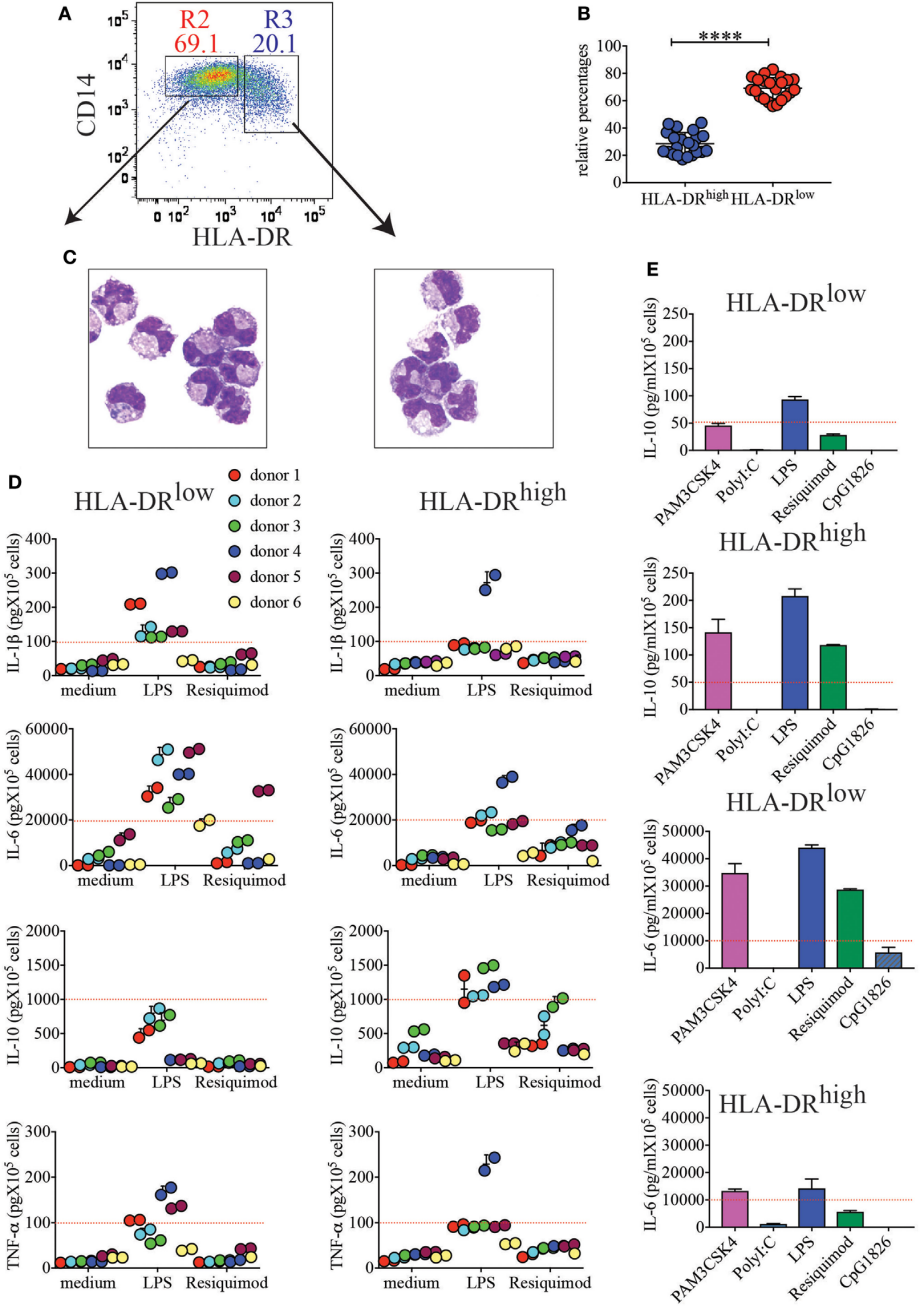
CD14^+^HLA-DR^high^ and CD14^+^HLA-DR^low^ cells, despite similar morphologies, respond differently to toll-like receptor (TLR) stimulation. **(A)** Representative sorting gate to separate CD14^+^HLA-DR^low^ and CD14^+^HLA-DR^high^ cells. **(B)** Relative percentages of HLA-DR^high^ to HLA-DR^low^ after purification with CD14 microbeads (*n* = 22). ****p < 0.0001. Significance was calculated by unpaired *T*-test. **(C)** Cytospin images of the two myeloid cell subsets. **(D)** Secretion of IL-1β, IL-6, IL-10, and TNF-α from HLA-DR^low^ or HLA-DR^high^ cells from different donors (*n* = 6). A total of 10^5^ cells were either left unstimulated (medium) or stimulated with LPS and resiquimod and cultured for 18 h prior to cytokine quantifications in supernatants. Each color represents a different donor and assays were run in duplicates. An arbitrary line was drawn to emphasize enhanced IL-1β and IL-6 secretions by HLA-DR^low^, while HLA-DR^high^ cells from most donors showed boosted IL-10 secretion. **(E)** IL-10 and IL-6 secretions by HLA-DR^low^ and HLA-DR^high^ placental cells in response to different TLR agonists. One representative donor of 2 tested with PAM3CSK4, PolyI:C, and CpG1826, in addition to LPS and Resiquimod, is shown. An arbitrary line was drawn to emphasize differential cytokine secretion.

In the attempt to identify the nature of HLA-DR^high^ cells in placenta, we stained each single lineage marker in combination with HLA-DR (not shown). HLA-DR^high^ cells expressed the lineage marker CD14 (Figure 2A), indicating that they represent a myeloid/monocytic or macrophagic cell subset. HLA-DR^high^ cells were always less represented than HLA-DR^low^ cells among CD14^+^ myeloid cells (average 17.1–41% *versus* 82.9–56.1%) (Figure 2B). When we FACS sorted the two subsets to high purity and stained them with an equivalent of the classical Wright-Giemsa stain, the morphology of the two subsets was surprisingly similar; both populations exhibited an indented nucleus and a cytoplasm containing many vacuoles, most likely corresponding to lipid droplets (Figure 2C).

Next, we tested the ability of these subsets to respond to TLR stimulation and to produce cytokines. We found that both subsets responded to some extent to TLR2, TLR4, and TLR7 stimulations (Figures 2D,E). However, in general, HLA-DR^high^ myeloid cells produced more IL-10 in response to both LPS and Resiquimod (Figure 2D), although statistical significance was only reached for LPS stimulation due to donor variation (Figure S1 in Supplementary Material). Conversely HLA-DR^low^ cells produced significantly more IL-1β and IL-6 in response to LPS (Figure 2D; Figure S1 in Supplementary Material). Both subsets produced TNF-α upon TLR4 engagement, and although more donors yielded higher TNF-α secretion from the HLA-DR^low^ subset (Figure 2D), statistical significance was not reached (Figure S1 in Supplementary Material). Neither subset responded vigorously to TLR3 or TLR9 stimulation (Figure 2E).

We conclude that, despite similar morphologies, the two subsets of myeloid cells identified here are prepared to respond to pathogens in different ways. HLA-DR^low^ cells might predominantly recognize Gram negative bacteria that may invade the placenta and they will produce an inflammatory environment to curb bacteria spreading. HLA-DR^high^ cells may recognize not only Gram negative bacteria but also ssRNA viruses or, most likely during uncomplicated normal term pregnancy, circulating fetal RNAs. By producing higher levels of IL-10 and lower levels of IL-6 and IL-1β, this cell subset may generate a tolerogenic milieu that avoids fetal rejection.

### Unbiased Gene Expression Profile of the Placenta Myeloid Subsets Further Corroborates the Tolerogenic Potential of HLA-DR^high^ Cells

**Figure 3:**
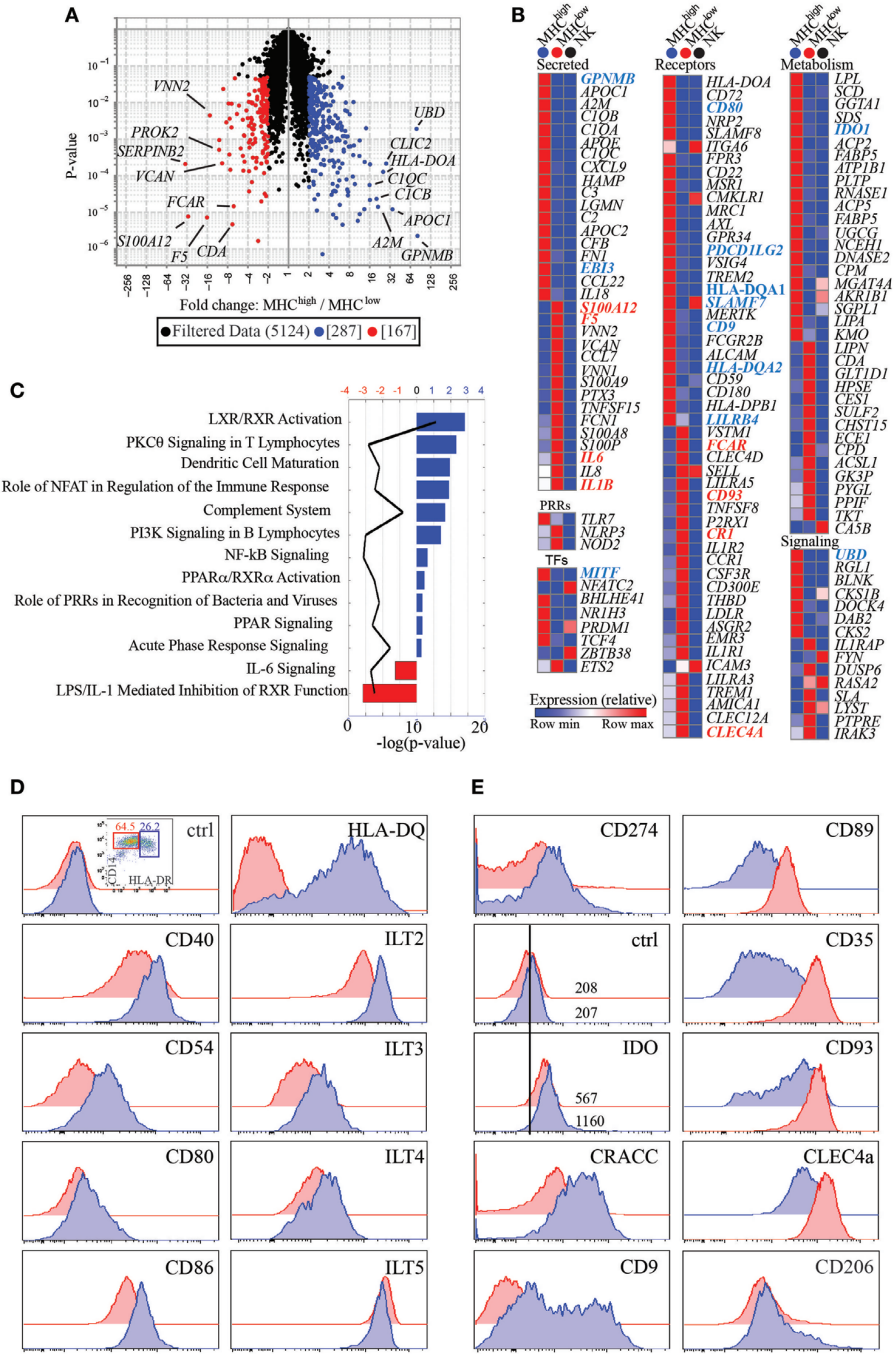
CD14^+^HLA-DR^low^ and HLA-DR^high^ have unique transcriptional signatures and differentially express cell surface receptors. **(A)** Volcano plot representing transcripts that are significantly and greater than two-fold expressed by HLA-DR^low^ (*n* = 3) (red) *versus* HLA-DR^high^ (*n* = 4) cells (blue). **(B)** Heatmaps of transcripts differentially expressed between HLA-DR^low^ and HLA-DR^high^ cells and categorized as secreted proteins, pattern recognition receptors (PRRs), transcription factors (TFs), cell surface receptors, and molecules involved in metabolism and cell signaling. Transcripts in bold blue (higher expression on HLA-DR^high^) or red (higher expression in HLA-DR^low^) indicate genes, which were subsequently validated by protein expression or RT-PCR. Human peripheral blood NK cells were used as comparison, with the rationale that having an innate but distant cell type may maximize our ability to identify genes differentially expressed between the two myeloid cell subsets. **(C)** Ingenuity pathway analysis networks with highest *z*-score generated from transcripts differentially expressed between HLA-DR^high^ (blue) and HLA-DR^low^ (red) cells. **(D)** Protein expression of costimulatory and antigen presenting molecules and inhibitory receptors of the immunoglobulin-like transcript family on HLA-DR^high^ (blue histograms) as compared to HLA-DR^low^ (red histograms) cells. **(E)** Increased protein expression of CD274, IDO, CRACC, and CD9 on HLA-DR^high^ cells and increased expression of CD89, CD35, CD93, and CLEC4A on HLA-DR^low^ cells. Both subsets express CD206 (mannose receptor) *ex vivo*.

In an attempt to better define the nature of the two myeloid subsets identified, we analyzed their gene expression signatures by global gene array profiling. We selected the genes that were significantly differentially expressed greater than two-fold between subsets (Figure 3A). As a control, we also compared expression of these genes with expression in a distant cell type, CD56^+^ NK cells from human peripheral blood. We identified 287 transcripts that were over-expressed in HLA-DR^high^ cells, while 167 were over-expressed in the HLA-DR^low^ subset. These genes encode several distinct classes of molecules, including secreted molecules, pattern recognition receptors (PRRs), transcription factors (TFs), cell surface receptors, and proteins involved in cell metabolism or in cell signaling (Figure 3B). Interestingly, HLA-DR^high^ cells expressed elevated levels of many complement factors, such as *C1QA, C1QB*, and *C1QC, C2* and *C3*, and *CFB*, suggesting that the complement pathway may play a pivotal role in pregnancy. In addition, they expressed high levels of *APOE* and *APOC2* and many metabolic genes involved in lipid handling and modification, such as *LPL, SCD, PLTP, FABP5, UGCG, NCEH1, SGPL1*, and *LIPA*. Moreover, HLA-DR^high^ cells expressed *EBI3*, also known as p28, a common subunit to the cytokines IL-27 and IL-35, both with known tolerogenic properties. The enzyme *IDO1* was also preferentially expressed on HLA-DR^high^ myeloid cells. This enzyme as well as a second isoform, *IDO2*, induces cell tolerance by degrading the essential amino acid tryptophan, thus limiting proliferation of antigen stimulated T cells (13). Intriguingly, HLA-DR^high^ cells also expressed the TF Blimp-1, which was shown to promote and sustain the tolerogenic function of DCs in female mice (14).

Consistent with the trend of HLA-DR^high^ cells producing higher levels of IL-10 upon Resiquimod stimulation (Figure S1 in Supplementary Material), these cells also expressed higher levels of *TLR7*. Conversely, HLA-DR^low^ cells had higher transcripts for genes involved in inflammation, such as the cytokines *IL6, IL8*, and *IL1B*. PRRs that are involved in recognition of bacterial components and inflammasome activation, such as *NOD2* and *NLRP3* were also more highly expressed. Noteworthy, only a few genes over-expressed on one of the two subsets were also expressed at high levels on peripheral blood NK cells [i.e., CRACC (*SLAMF7*), CD62L (*SELL*), *ITGA6*, and *ICAM3*].

Next, we performed IPA on genes with significant differences greater than two-fold between HLA-DR^high^ and HLA-DR^low^ cells. This analysis indicated that genes enriched in HLA-DR^high^ cells were linked to LXR/RXR signaling and PPRα/RXRα activation, nuclear receptors that mostly bind fatty acids or retinoids and control lipid metabolism. In addition, IPA linked genes enriched in HLA-DR^high^ cells to DC maturation and to the complement system. On the other hand, genes enriched in HLA-DR^low^ cells were connected to IL-6 signaling and to LPS/IL-1- mediated inhibition of RXR function, which promotes an inflammatory phenotype in macrophages (Figure 3C).

To functionally validate our data, we selected a few cell surface receptors for which antibodies were available and measured expression of these receptors on the two myeloid subsets. As shown in Figure 3D, costimulatory molecules such as CD40, CD54, CD80, and CD86, in addition to HLA-DQ, were higher in HLA-DR^high^ cells, in agreement with the DC maturation pathway identified by our IPA. In addition, several inhibitory receptors of the ILT family, such as ILT2 (*LILRB1*), ILT3 (*LILRB4*), and ILT4 (*LILRB2*) had higher expression on HLA-DR^high^, with ILT5 (*LILRB3*) being an exception, as it was higher on HLA-DR^low^ cells. CD274 (*PDCD1LG2*), a ligand for the inhibitory receptor programmed cell death 1 (PD1), had also higher expression on HLA-DR^high^, as did the enzyme IDO. Expression of IDO with a commercially available antibody was relatively low and an isotype-matched control was necessary to show a clear shift of the populations (Figure 2E). HLA-DR^high^ cells also expressed higher level of the CD2 family member CRACC (*SLAMF7*) (15), which mediate homotypic interactions and inhibit inflammatory cytokines in activated monocytes (16). Moreover, they expressed very high levels of CD9, a tetraspanin induced in human cells by TGFβ signaling (17), suggesting that HLA-DR^high^ cells may reside in a TGFβ rich environment or develop under the influence of TGFβ (Figure 3E).

In agreement with our gene array data, HLA-DR^low^ cells expressed higher levels of the IgA-specific Fc receptor CD89; the CR1 CD35; CD93, a receptor which promotes phagocytosis of apoptotic cells and of antibody and complement opsonized particles; and the lectin CLEC4A, also known as DICR (18), which binds carbohydrates, including some that decorate pathogens (19) (Figure 3E). Expression of these receptors supports the idea that this myeloid subset might be involved in bacterial recognition and removal of immunoglobulin or complement opsonized pathogens. Notably, CD206, the mannose receptor, was expressed on both myeloid subsets *ex vivo* (Figure 3E), suggesting that these cells are tissue specific and not blood derived monocytes, which *ex vivo* are CD206 negative (20).

**Figure 4:**
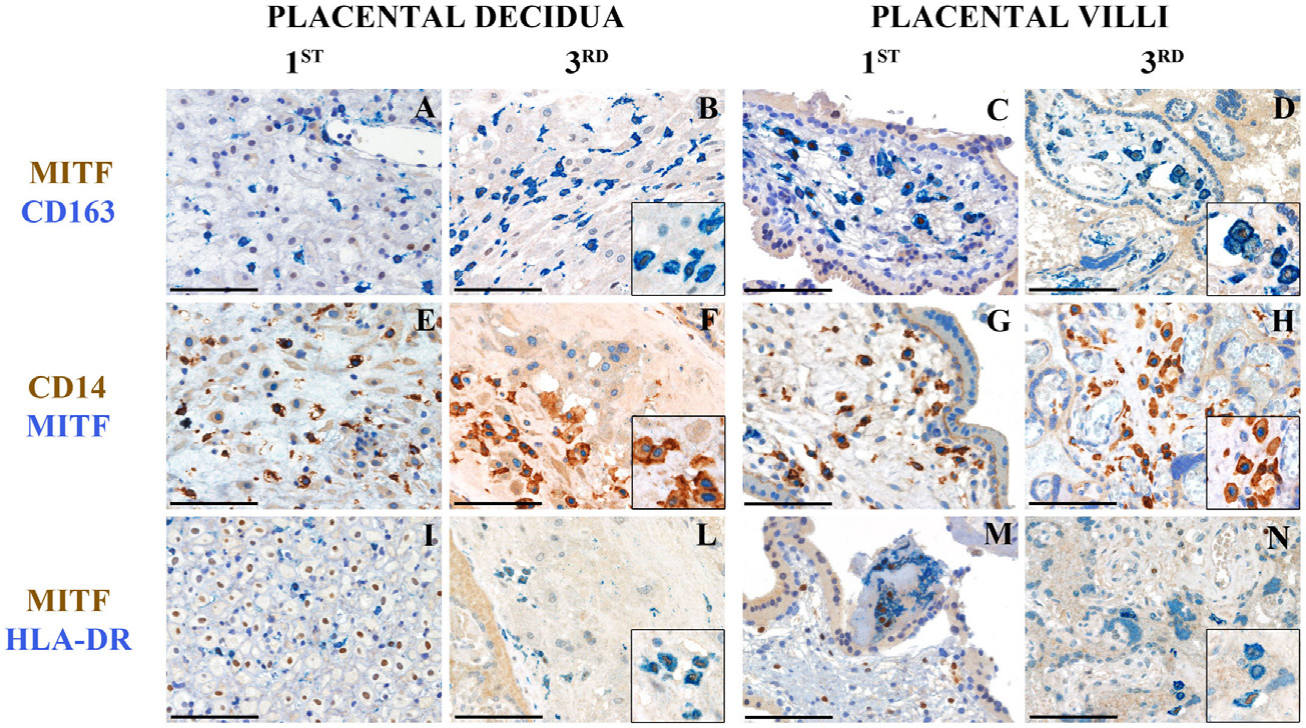
Tissue histology allows identification of MITF^+^CD14^+^ cells in human placenta at different stages of gestation. Sections are from first or third trimester normal placenta and stained as labeled. CD163^+^MIFT*^+^* myeloid are found in placental decidua and placental villi **(A–D)**. MIFT*^+^* myeloid cells that co-express CD14 **(E–H)** and HLA-DR **(I–N)** can be identified. Some of these cells are located within small fibrin deposits in the perivillous space **(M)** and within the decidua **(I–L)**. A variable level of expression of nuclear MIFT*^+^* is also observed in decidual cells at the 1st trimester **(I)**. Magnification 200× **(A–N)**; scale bare 100 μm. Inserts magnification 600×.

To localize the two cell types by immunohistochemistry, we took advantage of our microarray data showing higher expression of MITF on the HLA-DR^high^ subset. MITF is a TF of the MITF/TFE family involved in lysosomal biogenesis (21). We examined tissue sections from a set of archival placental tissue from the first and the third trimesters of gestation. As shown in Figure 4, many cells, at both time points, and, in both placental decidua and villi, stained positive for CD163 and CD14 (Figures 4A–H), two markers, which selectively identify myeloid cells of the monocyte/macrophage lineage. Some of these cells showed a clear nuclear-associated staining for MITF (Figures 4B,D,F,H, inserts) and seemed inter-dispersed among other CD163^+^ and CD14^+^ cells that stained negative for MITF, suggesting that the two myeloid cell types here identified did not have a distinct localization but may mingle in the same tissue microenvironment. By double staining with a MHC-class II-specific antibody, we found MHC-class II^+^MITF^+^ cells within small fibrin deposits in the perivillar space and within the decidua (Figures 4I–N). However, because of the broad MHC-Class II expression in human on many cell types, we cannot definitively conclude that those cells represent our HLA-DR^high^ myeloid cells.

**Figure 5:**
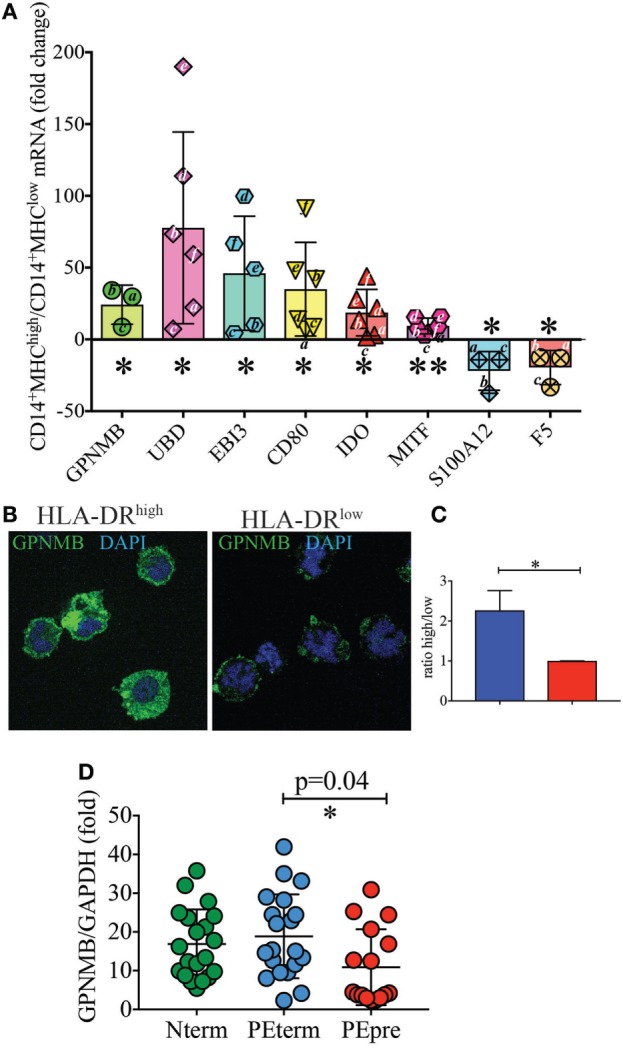
Validation of additional markers on HLA-DR^low^ and HLA-DR^high^ cells by RT-PCR and at protein level **(A–C)**. **(A)** Quantitative RT-PCR analysis of the indicated up- and down-regulated genes in CD14^+^ MHCII^high^ cells relative to expression in CD14^+^ MHCII^low^ cells, using *GAPDH* as a stable reference gene. Data are shown as mean ± SE, with symbols indicating results from individual placentas. A letter (i.e., a–c) was included within or next to the symbol to indicate matching donors. Statistical significance was determined by Student’s *t*-test. *p < 0.05 and **p < 0.005. **(B)** Representative GPNMB protein expression levels by immunofluorescence in CD14^+^MHCII^high^ and MHCII^low^ cells. FACS-sorted cells were co-stained for DNA (blue) and GPNMB (green) and the signal intensity of GPNMB determined using confocal microscopy. **(C)** Relative GPNMB signal intensity for the two cell types (*n* = 3). Statistical significance was determined by Student’s *t*-test. *p < 0.05. **(D)** Lower expression of GPNMB in placentas from pre-term preeclamptic pregnancies with severe complications. **(D)** *GPNMB* expression was determined by RT-PCR in basal plate tissue of placentas from women who had normal term pregnancy (Nterm, *n* = 20), pre-eclampsia and term delivery (PEterm, *n* = 20), and pre-eclampsia with severe complications and pre-term delivery (PEpre, *n* = 16). Values were normalized to GAPDH levels. Statistical significance was determined by ordinary one-way ANOVA.

To further confirm the differential expression profile of additional genes for which antibodies were not readily available, we performed real-time PCR experiments. Specifically, we aimed to validate *EBI3, GPNMB*, which is a direct downstream target of *MITF* (22, 23), *UBD*, the antimicrobial protein *S100A12* (24), and *F5*. *CD80, IDO*, and *MITF* were included as positive controls. Indeed, we found that *GPNMB, UBD*, and *EBI3* were expressed at significantly higher levels in HLA-DR^high^ than in HLA-DR^low^ cells (Figure 5A). We also tested GPNMB expression by immunofluorescent microscopy at the protein level, as this transcript was 80-fold more expressed on HLA-DR^high^ than on HLA-DR^low^. HLA-DR^high^ showed high expression of GPNM throughout the cytoplasm (Figure 5B), and quantification of the fluorescent signal by ImageJ analysis in the two populations was statistically significant (Figure 5C).

We conclude that a substantial portion of the data generated by our unbiased microarray analysis can be validated at the protein level and that pathways such as complement, apolipoproteins, inhibitory receptors/ligands, and immunoregulatory cytokines/enzymes are a major signature of HLA-DR^high^ myeloid cells in the term human placenta from uncomplicated pregnancies.

### Decreased GPNMB Expression May Represent a Prognostic Marker for Pregnancy-Associated Diseases

A precise function for GPNMB (also known as osteoactivin) in immune cells has not been reported. However, this protein, which is also released as soluble form, is highly expressed by some malignancies, including breast cancers and aggressive melanomas. Anti-GPNMB antibodies are currently in clinical trials for cancer (25, 26). Because of this link between GPNMB and cancer (27, 28), we hypothesized that GPNMB could be involved in maintaining a tolerant environment, which promotes a successful normal term pregnancy. We therefore sought to quantify GPNMB expression by real-time PCR in term placentas without pregnancy complications, placentas from term pregnancies complicated with pre-eclampsia and placentas from pregnancies with pre-term pre-eclampsia with severe features. As shown in Figure 5D, we did not observe significant differences between uncomplicated pregnancies and pregnancies with pre-eclampsia at term. However, despite large variations among samples, we detected a statistically significant difference in GPNMB expression between placentas of term pre-eclampsia and pre-term pre-eclampsia with severe feature, with the latter group showing reduced GPNMB expression.

### The PD1/PD ligand 1 (PDL1) Pathway Is Highly Represented in Normal Term Placenta

**Figure 6:**
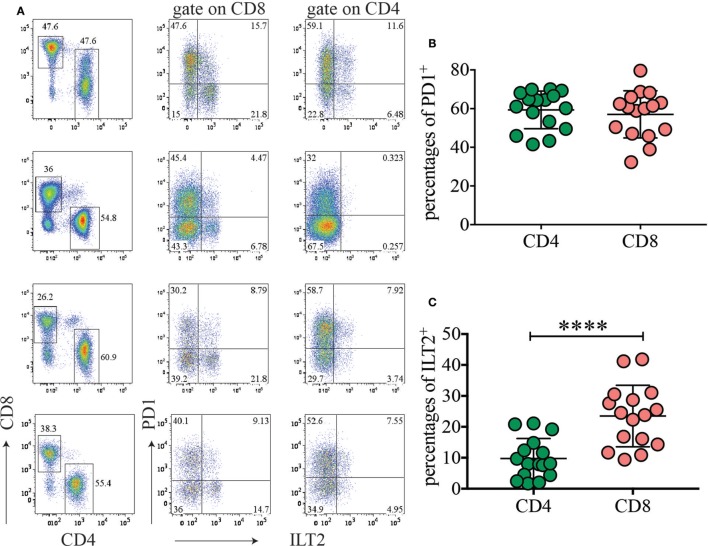
The inhibitory receptor programmed cell death 1 (PD1) is highly expressed among placental T cells. **(A)** Expression of PD1 and ILT2 (CD85J) on placental CD4 and CD8 T cells. Gate was applied on CD19^−^CD3^+^ T cells. Four representative donors out of 16 are depicted. **(B,C)** Percentages of PD1^+^ **(B)** and ILT2^+^ **(C)** CD4 or CD8 T cells in different donors (*n* = 16). ****p < 0.0001. Significance was calculated by Student’s *t*-tests.

As PD1 ligands, specifically CD274, had higher expression on HLA-DR^high^ myeloid cells, we hypothesized that this pathway could contribute to maintenance of tolerance during pregnancy. We therefore investigated expression of PD1 on T cells in normal term placenta. Interestingly, very high percentages of CD4 (41.5–70%) and CD8 (32.3–79.6%) T cells expressed high levels of PD1 *ex vivo* in all the donors tested (Figures 6A,B). To determine whether placenta T cells exclusively express PD1 or whether they also express other inhibitory receptors, we investigated expression of ILT2 (CD85J and LILRB1) which binds HLA-G present on syncytium-trophoblast (29) and also on our myeloid cell subsets (not shown). We found that a few CD4 (1.7–21%) and some CD8 (9.4–41.8%) expressed ILT2 (Figure 6C). However, expression of ILT2 was more variable among donors and never as prominent as PD1 expression.

This findings support the idea that the PD1/PDL1 axis might limit T-cell expansion driven by fetal alloantigen and may play a key role in human pregnancy and in establishing tolerance to the fetal allotransplant.

## Discussion

In this study, we identified two subsets of CD14^+^ myeloid cells that differ in terms of functional responses to TLR stimulation and cell surface receptors and have a very distinct transcriptional profile. A seminal study previously described two unique human decidual populations based on differential expression of CD11c (30). However, few of the genes identified in our study were also differentially expressed in those two subsets.

We find that our HLA-DR^high^ cells produce more IL-10 and express higher level of EBI3, a subunit of IL-27 and IL-35, two members of the IL-12 family of cytokines. Notably, IL-27 and IL-35 have both immunosuppressive and anti-inflammatory properties, though by different mechanisms (31–34). IL-27 is involved in induction of peripheral Foxp3^−^ Tregulatory 1 (Tr1) T cells, which are immunosupressive through the production of IL-10 (35–38). IL-35 instead is produced by conventional Foxp3^+^ Treg, which directly suppresses T-cell proliferation and differentiation (39). B cells can also produce IL-35 (40), which in turn induces other B cells to produce IL-10 (41, 42). From our data, it is not clear whether HLA-DR^high^ cells produce IL-27 or IL-35. However, when we looked in placental BP tissue for induced Tr1, based on expression of CD49b and LAG-3, as previously reported (43), we did not detect any substantial population of Tr1 among CD4 T cells. Furthermore, we did not find a significant population of CD4 T cells producing IL-10, when CD4 were maximally stimulated with PMA and Ionomycin (data not shown).

Interestingly, our HLA-DR^high^ myeloid cells expressed many factors of the complement cascade, as well as APOE and APOC2. It is well established that during pregnancy there is a systemic activation of the complement systems (44) and hyperlipoproteinemia. Increase in Apo-CII in serum is documented in late pregnancy (45). Our data now raise the intriguing possibility that placenta-associated myeloid cells may be a direct source of complement factors and apolipropoteins, whose levels increase in serum during pregnancy.

HLA-DR^high^ myeloid cells express PD1 ligands and other inhibitory receptors, such as ILTs (46). Importantly, PD1 is highly expressed on a large fraction of CD8 and CD4 cells on all donors tested, suggesting that the PD1/PD1 ligands’ axis is positioned to be an important player in establishing and maintaining tolerance in human pregnancy, similar to the role that PD1/PD1 ligands play in tumors (47, 48). Accordingly, blockade of PDL1 increases rejection of allogeneic concepti in mouse models (49). High expression of ILT2 and ILT4 on HLA-DR^high^ cells may also favor CD8 T cells with a Type-2 cytokine-secreting phenotype (50). In addition, because ILT2 and ILT4 binds to MHC class I, they might bind class I molecules in *cis* and sequester them from binding to TCR for antigen presentation to CD8 T cells (51).

We show that HLA-DR^high^ cells produce GPNMB, a soluble mediator expressed by highly aggressive tumors and now a putative target for cancer therapy (28). GPNMB expression on HLA-DR^high^ cells is likely driven by expression of the TF MITF, given the correlating expression pattern (22, 23). Although we did not observe any specific effects of soluble GPNMB in inhibiting proliferation of anti-CD3 stimulated T cells (data not shown), we cannot exclude that GPNMB has indirect immunosuppressive function on other cell types that in turn regulate T-cell proliferation and differentiation. We did observe that GPNMB expression was significantly lower in pre-term pre-eclampsia with severe feature. It has previously been shown that overexpression of GPNMB results in lower levels of pro-inflammatory cytokines, including IL-6 and IL-12 (52), which are elevated in pre-eclampsia (53). In other pathological conditions, such as in acute kidney injury, GPNMB functions as a negative regulator of inflammation by promoting IL-10 and TGF-β secretion (54). Therefore, a decrease in GPNMB expression in pre-eclampsia with severe features may represent a sign of worse disease. Alternatively, GPNMB may be upregulated with pregnancy progression. In the future differentiating between these two hypotheses will be important, as GPNMB levels in serum of pregnant women may be used as a potential biomarker to monitor the course and severity of pre-eclampsia.

Despite the fact that our HLA-DR^high^ subset bears some features of TGF-β imprinting, such as higher CD9 expression, we did not observe a specific niche within the decidual tissue or the villi in which MITF^+^CD163^+^CD14^+^ were preferentially located. On the contrary, these cells were interspersed with MITF^−^ CD163^+^CD14^+^ cells. This evidence may suggest that either TGF-β signaling differs between the two subsets or that one subset may convert to the other under yet unknown circumstances. Additional studies in animal models will be necessary to dissect these hypotheses.

In contrast to HLA-DR^high^ cells, HLA-DR^low^ cells seem prepared to mount anti-bacterial response based on their expression of pro-inflammatory factors such as many S100 proteins, IL-6, IL-1β, NOD2, and NLRP3. Collectively these factors are well-known drivers of acute inflammation following recognition of bacterial components in part via the inflammasome. This idea is also supported by greater expression of several endocytic receptors by HLA-DR^low^ cells, such as the C-type lectins DCIR, MCL, and MICL, which may recognize sugar moieties on pathogens. In addition, these cells express IgA receptors, such as CD89 and *ASGR2*, that may bind IgA opsonized bacteria at the mucosal surfaces. Finally, greater expression of S100A12 by HLA-DR^low^ cells, confirmed by RT-PCR, supports an antimicrobial role as this protein has large spectrum antibacterial/antifungal activity due to its zinc binding properties (24, 55).

More in depth analysis and animal models will be required to further dissect the function of the two myeloid subsets identified in our study. Nevertheless, our data clearly indicate the existence of at least two subsets of myeloid cells (monocytes/macrophages) in the BP of human normal term placentas, which may differ in their functional properties. One subset seems to exhibit a tolerogenic signature and to utilize immunosuppressive pathways that are often hijacked by tumors to avoid immune surveillance. The second subset seems to have more pro-inflammatory and defense properties and might be involved in protecting the fetus from exogenous pathogens. Single-cell RNA-seq experiments will also be required to verify whether diverse cell types are hidden in each subset, as recently shown for peripheral blood monocytes (56), or whether they represent homogenous populations with a unique transcriptional signature.

## Ethics Statement

This study was carried out in accordance with the recommendations of Washington University School of Medicine IRB committee with written informed consent from all subjects. All subjects gave written informed consent in accordance with the Declaration of Helsinki. The protocol was approved by the Institutional Review Board of the Washington University School of Medicine, St Louis, MO, USA.

## Author Contributions

MLC, BNC, and DMN were responsible for identifying placentas for collections and clinical information. MLC, EL, and MCe performed sortings and cell culture experiments. MLR performed gene array and IPA. MLC and MSL performed real-time PCR experiments. MB performed histology tissue stainings. WV supervised specimen collection for tissue histology, instructed histology tissue stainings, and interpreted histology data. DMN, MCo, and MCe conceived and supervised the study. All the authors analyzed and interpreted data. MCe wrote the manuscript with all co-authors contribution.

## Conflict of Interest Statement

The authors declare that the research was conducted in the absence of any commercial or financial relationships that could be construed as a potential conflict of interest.

## Supplementary Material

Figure S1 Compiled cytokine secretion data from six different donors to assess statistical significance. Median of duplicate values for each condition was calculated from the six donors in Figure 2A. Significance was calculated by ordinary one-way ANOVA Tukey’s multiple comparison test. **p* < 0.05, ***p* < 0.005, and *****p* < 0.0001.
