# Supplementary figures and images for: Two Distinct Myeloid Subsets at the Term Human Fetal–Maternal Interface
Source: Front Immunol. 2017 Oct 25;8:1357. doi: 10.3389/fimmu.2017.01357 (PMC5662895; doi:10.3389/fimmu.2017.01357)

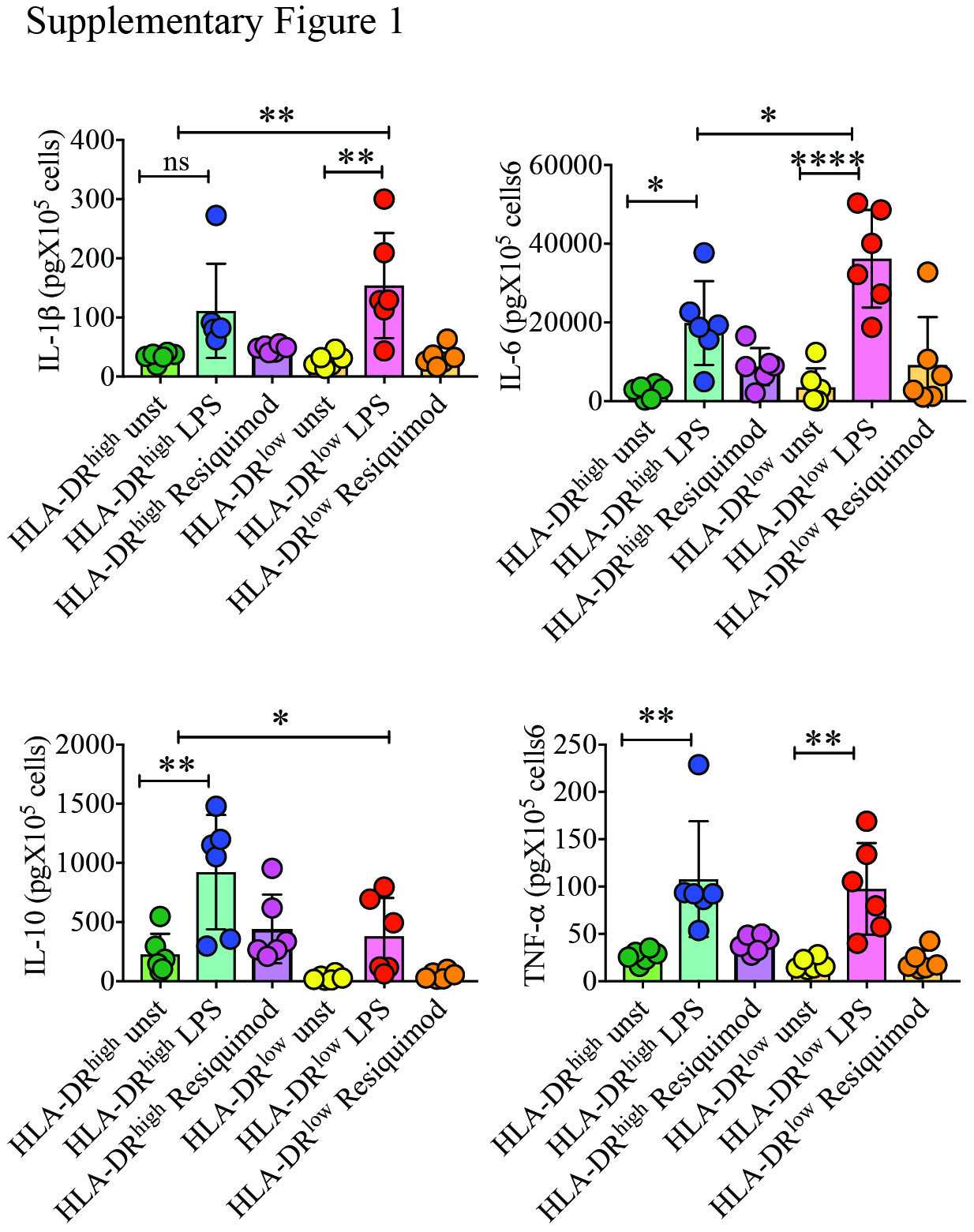

Supplement: Figure S1 — Compiled cytokine secretion data from six different donors to assess statistical significance. Median of duplicate values for each condition was calculated from the six donors in Figure 2A. Significance was calculated by ordinary one-way ANOVA Tukey’s multiple comparison test. *p < 0.05, **p < 0.005, and ****p < 0.0001. [file Image_1.JPEG]
